# Correction: Estimating the cooperativity of PROTAC-induced ternary complexes using ^19^F NMR displacement assay

**DOI:** 10.1039/d1md90045e

**Published:** 2021-12-20

**Authors:** Guilherme Vieira de Castro, Alessio Ciulli

**Affiliations:** Division of Biological Chemistry and Drug Discovery, School of Life Sciences, University of Dundee Dow Street Dundee DD1 5EH UK a.ciulli@dundee.ac.uk

## Abstract

Correction for ‘Estimating the cooperativity of PROTAC-induced ternary complexes using ^19^F NMR displacement assay’ by Guilherme Vieira de Castro *et al., RSC Med. Chem.*, 2021, **12**, 1765–1770, DOI: 10.1039/D1MD00215E.

The authors regret there was an error in their article on page 1768 (right column, 6 lines above Fig. 5). The sentence “CMP98 (*trans*–*cis*) which can only engage one VHL complex at a time, and the non-binding control CMP99 (*cis*–*cis*).” should read instead: “CMP99 (*trans*–*cis*) which can only engage one VHL complex at a time, and the non-binding control CMP98 (*cis*–*cis*).”

The Royal Society of Chemistry apologises for these errors and any consequent inconvenience to authors and readers.

## Supplementary Material

